# Pros and cons for the evidence of adaptive non-shivering thermogenesis in marsupials

**DOI:** 10.1007/s00360-021-01362-0

**Published:** 2021-04-15

**Authors:** Martin Jastroch, Elias T. Polymeropoulos, Michael J. Gaudry

**Affiliations:** 1 Department of Molecular Biosciences, The Wenner-Gren Institute, Stockholm University, 10691 Stockholm, Sweden; 2 Institute for Marine and Antarctic Studies (IMAS), University of Tasmania, Hobart, TAS 7001 Australia

**Keywords:** Marsupials, Adaptive non-shivering thermogenesis, Brown adipose tissue, Endothermy

## Abstract

The thermogenic mechanisms supporting endothermy are still not fully understood in all major mammalian subgroups. In placental mammals, brown adipose tissue currently represents the most accepted source of adaptive non-shivering thermogenesis. Its mitochondrial protein UCP1 (uncoupling protein 1) catalyzes heat production, but the conservation of this mechanism is unclear in non-placental mammals and lost in some placentals. Here, we review the evidence for and against adaptive non-shivering thermogenesis in marsupials, which diverged from placentals about 120–160 million years ago. We critically discuss potential mechanisms that may be involved in the heat-generating process among marsupials.

## Introduction

Endogenous heat production enables many vertebrates to maintain body temperatures above environmental temperature, supporting higher enzymatic functions and bestowing certain advantages (e.g. greater activity through sustained muscular/locomotor function in colder ambient temperatures, higher brain functions, increased success for reproduction and growth, opportunities for niche expansion) despite increased energetic demands. Sensing of environmental temperature and distinctive adjustments of heat output are prerequisites that enable sustained high body temperatures. Heat production is supported by basal metabolism, and for thermoregulatory purposes, facultative metabolism can be recruited, allowing some vertebrates to maintain a homeothermic cellular environment. Facultative thermogenesis may be upregulated via muscle activity during locomotion, but involuntary skeletal muscle tremors during shivering are another immediate source of thermoregulatory heat production by increasing metabolic turnover rates. Birds and mammals achieve sustained high body temperatures, but endothermic homeothermy also appears sporadically in other vertebrate clades, presumably providing an edge for performance. Some lamnid sharks and teleosts (e.g. the bluefin tunas and the opah) maintain a homeothermic endothermic environment isolated by counter-current systems and supported by large body size (Carey and Teal 1966, 1969; Wegner et al. 2015). Billfishes possess a modified retro-ocular muscle that serves as a brain heater organ improving vision and thus predation (Carey 1982; Fritsches et al. 2005). In reptiles, there are reports on reproduction-related maintenance of high body temperature in pythons and tegu lizards (Brashears and DeNardo 2013; Harlow and Grigg 1984; Tattersall et al. 2016) as well as high body temperatures in sea turtles that have been related to gigantism, countercurrent heat exchangers, and insulative adipose tissue (Casey et al. 2014; Davenport et al. 2009; Frair et al. 1972; Greer et al. 1973), fueling theories of independent evolutionary origins of endothermy. The mechanisms of endothermy, homeothermy, and thermogenesis, however, have been best studied in placental mammals. In placentals, shivering has always been regarded as an immediate form of heat production that is replaced by non-shivering thermogenesis (NST), if possible. Indeed, NST is thought to be advantageous by maintaining the insulative boundary layer of air surrounding the body, freeing skeletal muscles to do other work. On the hunt for the source of NST in placental mammals, brown adipose tissue (BAT) has been recognized as the primary contributor in seminal work since the 1960s (Smith 1961), followed by surveys showing morphological evidence for brown fat in a wide array of placental mammals (Rowlatt et al. 1971) including adult humans (Heaton et al. 1972). The central-nervous control of BAT through postganglionic noradrenaline (NA) release was shown by Foster and Frydman (1978) and the important heat-catalyzing mitochondrial protein, uncoupling protein 1 (UCP1), was first biochemically described by Nicholls and colleagues (Heaton et al. 1978), before eventually being cloned in 1985 (Aquila et al. 1985; Bouillaud et al. 1985).

The molecular knowledge on BAT has been almost exclusively gathered from eutherian mammals, mostly rodents, from the Northern hemisphere. BAT is morphologically unique due to its dense vascularization with multilocular fat droplets dispersed throughout the cytoplasm, supplying the required fuels for the combustion process, and massive amounts of mitochondria, providing the oxidative capacity for heat generation. BAT warms in particular the body core since the depots are distinctly located near critical organs (e.g. spinal cord, heart, kidneys), and major blood vessels (e.g. Sulzer’s vein) transport warm blood directly to the heart to distribute heat to the rest of the body. Since shivering is reliant on musculature located in the periphery of the body, much of this heat is presumably dissipated to the surrounding environment without substantial contributions to warming the body core. UCP1 knockout mice require 60% more energy for arousal from fasting-induced torpor than wildtype mice (Oelkrug et al. 2011), underscoring the energetic advantage of UCP1-mediated NST.

Collectively, adaptive NST among eutherian mammals has been defined to require BAT, and the identification of BAT has been broadly accepted as evidence for NST. Thus, for the identification of NST in non-placental lineages, researchers were guided by previous studies in BAT-positive placental mammals, searching for typical BAT morphology, noradrenalin-induced thermogenesis, and the presence of UCP1. In particular, UCP1 has been claimed to be pivotal for adaptive NST, based on studies in the UCP1 knockout mouse (Cannon and Nedergaard 2004; Golozoubova et al. 2001, 2006). The importance of BAT for mammalian thermogenesis and evolution has been highlighted by the suggestion that BAT-thermogenesis and its unique protein, UCP1, provided mammals with an evolutionary edge that promoted the success of the lineage (Cannon and Nedergaard 2004). From a phylogenetic perspective, however, this statement assumed that BAT and UCP1 must have been present in the common ancestor of all mammals, which comprise the extant groups of monotremes, marsupials, and placentals. The presence of thermogenic BAT in marsupials and monotremes, however, is still matter of debate and thus, the origin of BAT remains experimentally undelineated.

Monotremes are egg-laying mammals that shared a common ancestor with a stem-marsupial-eutherian ~ 200 million years ago. Marsupials diverged from a stem-eutherian between 120 and 160 million years ago, have a pouch or pouch-region to embed underdeveloped young to maintain a stable thermal environment. Extant marsupials are found mainly on the Australian continent, with fewer species on the American continent. Genetic data suggest that they all descend from South American ancestors and the separation between American and Australian marsupials has been dated to ~ 80 million years ago (Renfree et al. 2011). Similar to other dating attempts, this number has to be taken with caution as geological data suggest the break-up of Gondwana, separating South America and Australia, at a much earlier time-point (Storey 1995).

The matter of BAT biology has been voluminously reviewed for eutherian mammals in 2004 (Cannon and Nedergaard 2004), yet the controversial presence of BAT in monotremes and marsupials was largely neglected and insufficiently covered, highlighting only a few scattered studies. Already at that time, there had been a substantial body of investigations on the presence of NST, BAT and UCP1 in marsupials and monotremes, although with highly ambiguous results. Clarification on the presence of functionally thermogenic BAT and UCP1 is, however, not only important for marsupial and monotreme biology and ecophysiology, but also of paramount interest for the understanding of thermogenic evolution and the existence of UCP1-independent thermogenic mechanisms.

## The BAT-centric approach to investigate NST in marsupials and monotremes

The historically imprinted definition of NST, sometimes also referred to as “classical NST”, demands adaptivity to environmental fluctuations (Cannon and Nedergaard 2004). Several experimental observations have been considered as arguments for NST. Smaller eutherian mammals (< 10 kg) show a metabolic response to NA (Heldmaier 1971; Oelkrug et al. 2015), which corresponds to the presence of BAT (Rowlatt et al. 1971), its quantity and more or less to UCP1 content. The mitochondrial oxidative capacity determines thermogenic potency in BAT, while UCP1 serves as the valve that allows metabolic and futile cycles to run at faster paces. Thus, the amount of UCP1 may only correlate weakly with the heat output of the tissue or peak rewarming rates (Oelkrug et al. 2014; Wang et al. 2021). In any case, the elegant BAT-mediated NST mechanism, that has been so well described in eutherians, has driven a BAT-centric approach to investigate its state of conservation throughout the mammalian class (i.e. also in non-eutherian mammals) and the experimental results have often been generalized to either claim or dismiss the presence of adaptive NST in non-eutherians.

## Physiological evidence for and against adaptive NST in marsupials

In Table 1, we summarize potential mechanisms of NST among marsupials (some of which have yet to be examined), the experimental techniques used to investigate them, as well as the types of data that are typically collected in support of these mechanisms. We also list a number of caveats that may confound the investigation of such mechanisms. One overarching caveat may be that marsupials generally occupy more mild climates in comparison to many eutherian mammals (e.g. rodents) that display a heavy reliance on BAT-mediated thermogenesis during the cold winter months. Thus, less extreme seasonality could be exhibited, hindering our detection of adaptive NST. Indeed, at least some phylogenetically basal eutherians (e.g. *Elephantulus myurus*—members of the afrotheria), have also been found to display mild seasonal differences in adaptive NST (Mzilikazi et al. 2007).

**Table 1:**
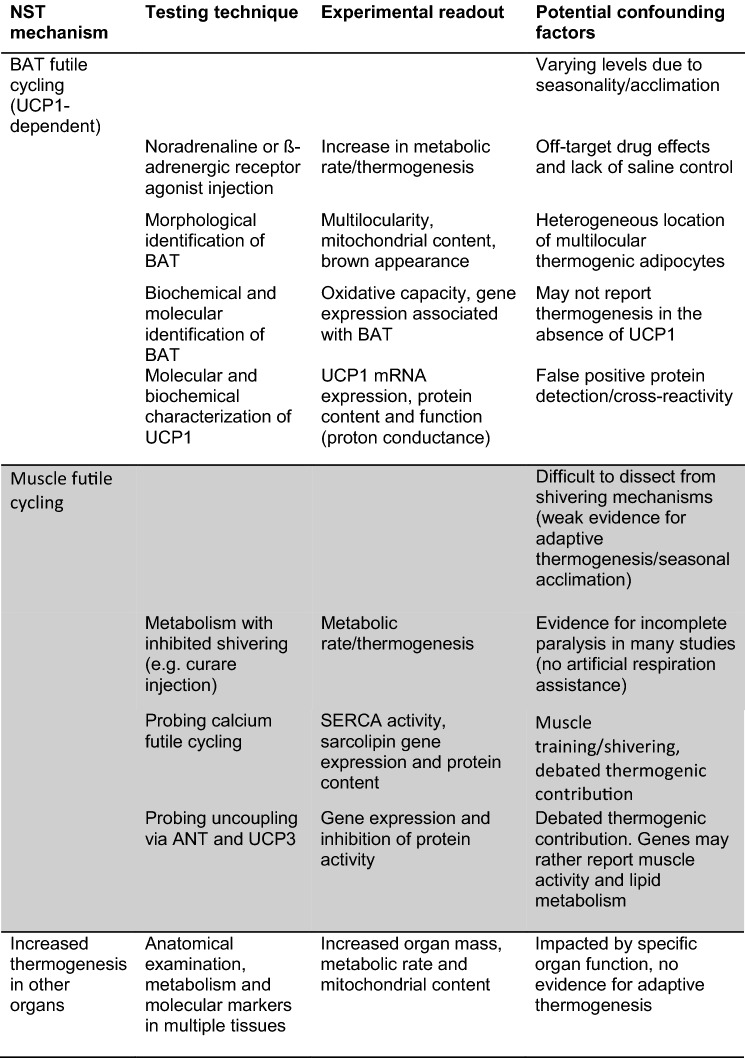
Potential NST mechanisms in marsupials, typical techniques used for their investigation, the experimental readouts and possible caveats

Hinting towards the possibility of adaptive NST in marsupials, stripe-faced dunnarts (*Sminthopsis macroura*) upregulate heat production by 27% when cold-acclimated from 26 to 16 °C for 16 days (Geiser et al. 2003). It is worth noting, however, that the mechanism of increased heat production was unexamined in this study and could be at least partly due to shivering. Since the classic activator of NST in eutherians has been NA, several studies have also administered NA to marsupials in search of metabolic response. For instance, Tasmanian devils (*Sarcophilus harrisii*; Kabat et al. 2003a), Tasmanian bettongs (*Bettongia gaimardi*; Rose et al. 1999), and eastern barred bandicoots (*Perameles gunnii;* Rose and Ikonomopoulou 2005) all show an enhanced metabolic rate (MR) to NA following cold acclimation. Bennett’s wallaby (*Macropus rufogriseus rufogriseus*) joeys, however, decrease MR or show no change in response to NA injection when ≤ 250 g in body size, but do show an increased MR at 400 g (Loudon et al. 1985). Interestingly, the latter is a developmental stage of about one month before leaving mother’s pouch. At this time-point, Loudon et al. (1985) were also able to identify a putative interscapular brown fat structure by electron microscopy displaying multilocularity and high mitochondrial density.

By contrast, several species, even after cold acclimation, do not increase MR following NA injection, including the gray short-tailed opossum (*Monodelphis domestica*; Dawson and Olson 1988), the brown antechinus (*Antechinus stuartii*; Reynolds and Hulbert 1982), and the monito del monte (*Dromiciops gliroides*; Cortés et al. 2014). The NA-response documented in fat-tailed dunnarts (*Sminthopsis crassicaudata*; Clements et al. 1998) is not elevated by cold-acclimation (Polymeropoulos et al. 2012a), similar to what is found for Chilean mouse-opossums (*Thylamys elegans*; Opazo et al. 1999). In *T. elegans*, however, Opazo et al. (1999) concluded that while the rates of rewarming from torpor do not differ between cold and warm acclimated individuals, the energetic demands of rewarming were reduced with cold acclimation. The reduced energetic demands were interpreted as an improvement in shivering efficiency, though this has yet to be directly examined. Nicol et al. (2009) compared rewarming rates from the hibernation of the short-beaked echidna (*Tachyglossus aculeatus*), a monotreme that does not possess BAT, with the alpine marmot (*Marmota marmota*), a eutherian that is known to rely heavily upon BAT. This study concluded a significant contribution of BAT resulting in doubled rewarming rates in the marmot, which is in line with Oelkrug et al. (2011) who demonstrated lower rewarming rates in UCP1 knockout vs. wildtype mice.

The “classical” NA response in eutherians is typically induced through the action of the ß3-adrenergic receptor localized to the plasma membrane of brown adipocytes. To address the responsible noradrenergic receptor, Nicol (1978) tested the effect of isoprenaline, adrenaline, and noradrenaline on MR in the long-nosed potoroo (*Potorous tridactylus*). While all induced a metabolic response, isoprenaline, a non-selective beta-adrenergic receptor agonist, was the most potent inducer of increased MR (i.e. thermogenesis). By contrast, NA was the least effective, requiring a fivefold higher dose (9 µmol/kg) than would be expected for an equally sized placental mammal to achieve the maximal response. Also, potoroos did not display differing NA responses following at least 7 weeks of exposure to 20 °C compared to wild-caught animals that had overnight ambient temperatures around 5 °C, suggesting a lack of adaptive NST. Indeed, Nicol (1978) found no morphological evidence for BAT upon dissection and visual inspection of the potoroos. Notably, Nicol et al. (1997) pointed out that the high NA dose may have been problematic as it possibly induced cardiovascular effects and is lethal for other small mammals like rabbits (Nicol 1978; Nicol et al. 1997).

**Fig. 1:**
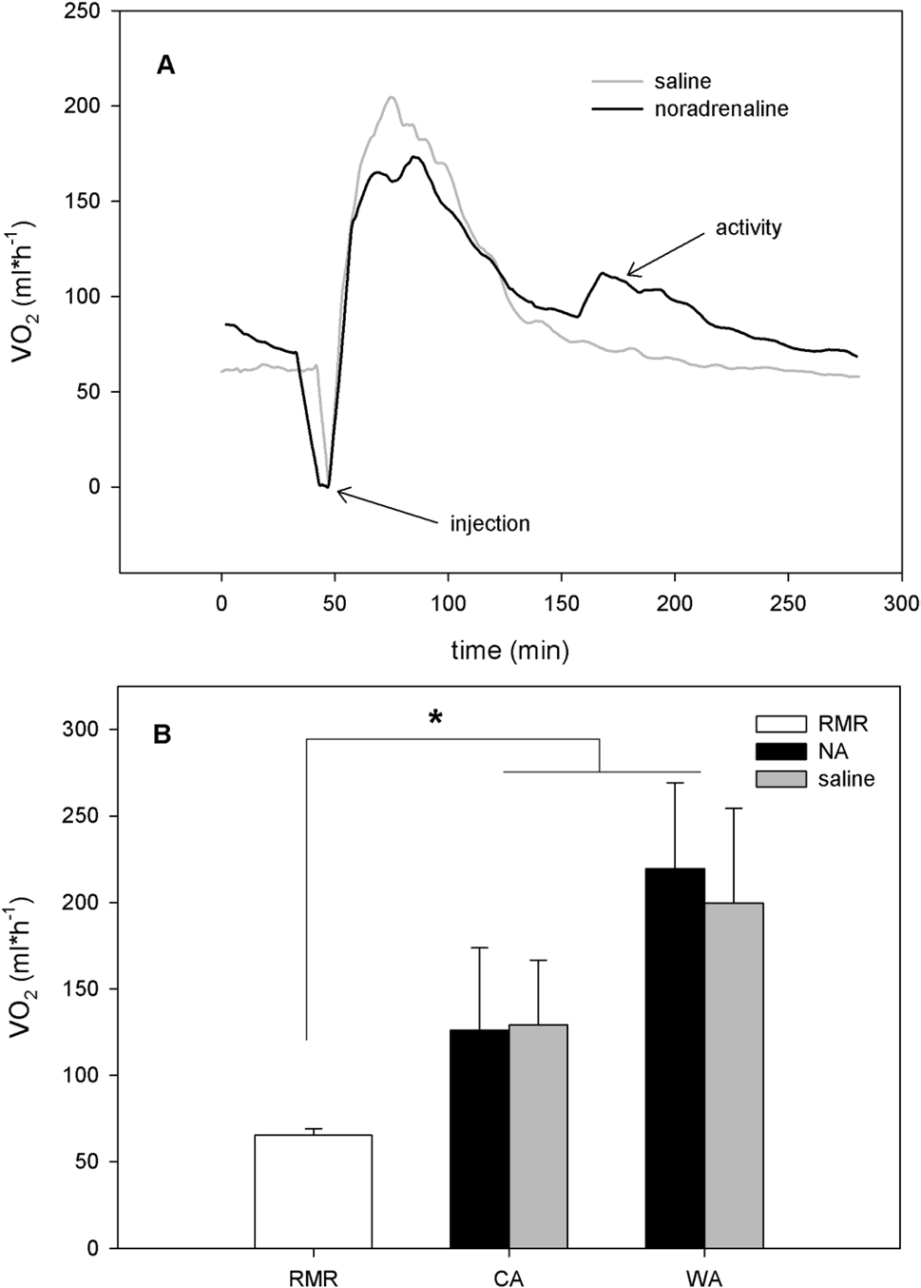
Metabolic assessment of NST capacity of adult *M. domestica* suggest neither adrenergic control, nor adaptivity. Rates of oxygen consumption (*V*O_2_, ml h^−1^) of adult *M. domestica* was measured using indirect open flow respirometry, as described in detail in Polymeropoulos et al. (2012b), at an ambient temperature of 30 °C. To determine the NST capacity, cold (CA, 12 °C, *n* = 3) and warm (WA, 26 °C, *n* = 3) acclimated animals (exposure time 3 weeks) were injected with either noradrenaline (NA) or saline in a randomized order. NA dosage was determined as follows: mg kg^−1^ dosage (mg*kg^−1^ body mass) = 6.6* body mass ^−0.458^ for each individual and NST capacity was calculated according to Heldmaier et al. (1971). Injection of either NA or saline occurred after RMR levels were established initially and the *V*O_2_ response was measured until RMR levels were re-established post injection. **a** Exemplary trace of changes in *V*O_2_ in *M. domestica* after NA (black line) and saline (grey line) injection. **b** NST capacity and RMR in WA and CA *M. domestica.* Values presented are mean ± S.D. There was no statistical difference within or between groups comparing the response of NA to saline injection with either being significantly elevated in acclimation groups compared to RMR, as determined by 2-way repeated measures ANOVA with the level of significance set to *p* < 0.05. RMR did not differ between groups and were therefore presented as a single value

There are various experimental aspects to NA tests that may be confounding factors. The use of saline control injections is required to control for animal handling stress but has not been applied in all studies. Unpublished data of our laboratory show in adult *Monodelphis domestica* elevated metabolic rates upon NA-injections, which can be fully addressed to handling stress as these increases are also seen with the saline control (Fig. 1). These observations are in line with previous data showing a lack of NA-induced thermogenesis in *M. domestica* (Dawson and Olson 1988; Nicol et al. 1997). While others suggested the existence of adaptive thermogenic pathways in *M. domestica* (Dawson and Olson 1988), these appear not to be under adrenergic control, given that the magnitude of the NA-response is not affected by acclimation temperature (Fig. 1). Using NA to examine marsupial NST may be hampered by unintended cardiovascular effects of NA that could lead to an increased MR through vasoconstriction or affecting rate and contractile strength of the heart beat (Nicol et al. 1997; Ye et al. 1995). While the cardiovascular response to NA is likely negligible in eutherian mammals that display classical BAT-mediated metabolic responses, it is possible that modulation of cardiovascular activity is predominantly responsible for the observable MR increase in species that lack BAT (Nicol et al. 1997). Thus, NA injection alone cannot confirm the presence of classical BAT-mediated NST. Nicol et al. (1997) addressed this shortcoming by testing selective agonists for the ß3-adrenoreceptor (ICID7114 and BRL35135) in the brushtail possum (*Trichosurus vulpecula*), the gray short-tailed opossum, and Bennett’s wallaby. These agonists did not induce a response, nor did NA in any of the marsupial species examined. Experiments using a variety of adrenergic agonists are not entirely decisive to judge the lack of BAT in marsupials, as it is conceivable that BAT-mediated responses are not under the control of the ß3-adrenoreceptor. For example, this is the case in guinea pigs, which display BAT, but lack a response to various ß3-adrenergic agonists (Himms-Hagen et al. 1995). Other marsupials such as the Tasmanian bettong are metabolically responsive to the ß3-adreneric receptor agonist BRL 37344 (Rose et al. 1999). Yet, in this species, NA-induced NST responses have been attributed to α1-adrenoreceptors in skeletal muscle (Ye et al. 1996)**.** These contradictory findings highlight the need for an array of histological, immunological and molecular techniques when searching for classical BAT-mediated NST marsupials.

## Morphological evidence for and against BAT in marsupials

The presence of BAT has also been controversial in marsupials with many anatomical studies reporting that marsupials lack the tissue altogether. In histological examinations of multiple species, Rowlatt et al. (1971) examined one newborn monotreme (the short-beaked echidna; *T. aculeatus*) as well as seven newborn marsupial species (31 individuals total) and found no evidence for BAT in the neck and axilla regions. Similarly, Hayward and Lisson (1992) specifically targeted marsupial species from cold regions and of smaller body size in an attempt to maximize the possibility to identify BAT. Examining 83 individuals in total, comprising 38 species (including 2 monotremes) and 16 extant families, they stated no detection of BAT using light, electron, and fluorescence microscopy, but noted the presence of multilocular white fat following food deprivation or cold stress.

Besides the aforementioned evidence for BAT (Loudon et al. 1985), Hope et al. (1997) compared the morphology of adipose tissue from the interscapular and tail regions of *S. crassicaudata* by electron microscopy. While the majority of the interscapular adipocytes were occupied by a single large lipid droplet, some cells were multilocular with smaller droplets. Interestingly, the interscapular depot was noticeably more vascularized and mitochondria-dense compared to the adipocytes from the tail depot. Jastroch et al. (2008) also depicted ‘brownish’ adipose tissue in the interscapular region of *S. crassicaudata* that becomes visually more brownish upon cold acclimation.

## Molecular evidence for and against UCP1 in marsupials

UCP1 is the key protein to catalyze thermogenesis in BAT. However, immunological identification of marsupial UCP1 should be considered experimentally flawed if heterologous antibodies were used. This approach, however, has been frequently published in the pre-genomic era for non-model organisms, due to the lack of more convincing methodologies such as genomic and RNA sequencing. Briefly summarizing the history of attempted immunological UCP1 protein detection in marsupials: the presence of UCP1 was claimed when probing the interscapular adipose depot of *S. crassicaudata* using antibodies against ground squirrel (Hope et al. 1997). The absence of UCP1 was claimed when probing adipose tissue of Tasmanian bettong and Tasmanian devil (Kabat et al. 2003a, b) but, considering their use of an unspecified eutherian UCP1 antibody, this is rather an absence of evidence than evidence of absence.

Probing for *UCP1* mRNA expression represents a more advanced approach for the presence and expression level of *UCP1* that can be eventually proven with sequencing, and if no obvious premature stop codons are found in the coding sequence as it is the case for horse *UCP1* (Gaudry et al. 2017), a functional protein should be present.

**Fig. 2:**
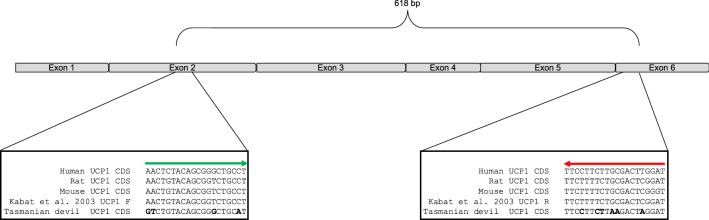
Schematic of *UCP1* coding sequence and inset alignments illustrating priming location of published primers to detect marsupial *UCP1* from the eutherian mammal (human, rat, mouse) consensus, which were used in an attempt to amplify *UCP1* mRNA of the Tasmanian devil (Kabat et al. 2003a) and the Tasmanian bettong (Kabat et al. 2003b) by RT-PCR. Due to the considerable sequence divergence between eutherian and marsupial *UCP1*, several nucleotide mismatches with respect to Tasmanian devil *UCP1* (Gaudry et al. 2017) are present and highlighted in bold, suggesting experimental failure regarding the negative PCR results (Kabat et al. 2003a, b) Note also that the authors expected an amplicon length of 213 bp (Kabat et al. 2003b), while the actual amplicon size should be 618 bp

Using Northern blots, Rose et al. (1999) did not detect *UCP1* mRNA in the interscapular adipose tissue of the Tasmanian bettong, even after two weeks of cold exposure at 4 °C, using a probe generated from the rat *UCP1* cDNA sequence. These results should be taken with caution, however, considering the substantial sequence divergence between *UCP1* of marsupials and eutherians (Gaudry et al. 2018; Hughes et al. 2009), which may fail to detect *UCP1* mRNA as was the case in *S. crassicaudata* (Jastroch et al. 2008). Further experimental caveats can be caused by hybridization conditions, rendering the use of cDNA probes ambiguous. A conserved 27 base pair probe (Brander et al. 1993) also revealed no *UCP1* expression in the Tasmanian bettong (Rose et al. 1999). Although the absence of *UCP1* in the Tasmanian bettong and Tasmanian devil was supported by RT-PCR trials (Kabat et al. 2003a, b) these experiments were performed using primers designed from a eutherian consensus sequence of human, rat, and mouse *UCP1* and not validated with a marsupial *UCP1* positive control. Indeed, a comparison of the primers used by Kabat et al. (2003a, b) to the *UCP1* coding sequence retrieved from the subsequently sequenced Tasmanian devil genome reveals numerous nucleotide mismatches (Fig. 2) that could preclude the amplification of the targeted fragment altogether.

Unambiguous proof for the existence of the *UCP1* orthologue in marsupial was first discovered in the marsupial *M. domestica* by genomic detection and subsequent sequencing (Jastroch et al. 2008). The genomic sequencing results also confirmed the conserved synteny of the 5′-*TBC1D9*-*UCP1*-*ELMOD2*-3′ gene cluster. *M. domestica UCP1* mRNA was detected by RT-PCR in pectoral fat of 70-day old animals. The expression levels in these experimental animals were, however, negligible as whole body in situ hybridization did not detect *UCP1,* but did indicate *UCP2* expression, a paralogous protein of UCP1 with an undefined function. Northern blotting did not reveal any *UCP1* mRNA detection and regulation in the interscapular fat of young adolescent opossums (Jastroch et al. 2008). In *Antechinus flavipes*, a dasyurid species from subtropical rainforests, Northern blots with a homologous probe on the interscapular fat depot similarly revealed no UCP1 expression. However, in another dasyurid, *S. crassicaudata*, this technique demonstrated cold-induced *UCP1* mRNA expression in the interscapular adipose tissue, the same anatomical region of the primary BAT depots in rodents (Jastroch et al. 2008).

While UCP1 expression appears to be specifically localized to adipose tissue in marsupials (at least for *M. domestica* and *S. crassicaudata*), the function of marsupial UCP1 as an uncoupler of mitochondrial respiration has yet to be determined. Interestingly, it has been suggested that accelerated evolution in a stem eutherian ancestor resulted in considerable sequence divergence prompting a novel function in eutherians (Hughes et al. 2009; Saito et al. 2008). The eutherian *UCP1* enhancer box, containing a number of putative transcriptional response elements (PPRE, TRE, CRE), is absent in marsupials (Gaudry and Campbell 2017; Jastroch et al. 2008) hinting that differences exist in the transcriptional regulation of the gene between marsupials and eutherians. For the understanding of the origin of BAT thermogenesis and NST, it will be pivotal to understand whether marsupials are able to uncouple the respiratory chain similarly to eutherian UCP1. As marsupials are not standard experimental animals in most laboratories, functional studies on the ectopically expressed protein may prove useful and the increasing sequence information of the orthologues of diverse species could enable the reconstruction of ancestral proteins that reveal key structure–function modifications.

## Are alternative sources of adaptive NST relevant in marsupials?

There are several ways in which marsupials may be generating heat alternatively of UCP1-dependent thermogenesis, most prominently shivering. Schaeffer et al. (2003) demonstrated that in *M. domestica*, 8 weeks of cold is sufficient to increase relative heart size and mitochondrial volume densities in skeletal muscle, leading to greater oxidative capacity, thermogenic potential, and indicating enhanced shivering activity. Similarly, however, relative liver mass increases by 48% in cold-acclimated *M. domestica* and hepatocyte mitochondrial volume is also significantly higher (Villarin et al. 2003). Greater liver, kidney, and caecum dry mass has also been reported in 4 weeks cold exposed *T. elegans* (Nespolo et al. 2002). The basal metabolic rate of *T. elegans* was ~ 30% higher in cold-acclimated compared to warm-acclimated individuals and it was calculated the increased size of the intestines and kidneys accounts for ~ 70% of this difference. Despite the lack of coherent responses to NA, substantial increases in thyroid hormone secretion were reported following six to eight weeks of cold exposure in *Antechinus stuartii,* which were not found in control mice, suggesting a role of thyroid hormone in marsupial endothermy and adaptive thermogenesis (Withers and Hulbert 1988). Indeed, seasonal thyroid hormone fluctuations in the Tammar wallaby reveal elevated levels in winter months with lower levels in the summer (Kaethner and Good 1975) though more thorough examinations are required to determine exactly how thyroid hormone may be influencing endothermic responses. All these factors are suggestive of augmenting overall metabolism that would result in greater heat output. However, whether organs other than adipose tissue and skeletal muscle can contribute to adaptive thermoregulation is unknown. The increases in the size of metabolically active tissues in the cold may contribute to systemic heat production, but may not allow for rapid adaptive increases of heat output without affecting organ function.

Besides enhanced shivering, others would argue for the existence of NST-mechanisms in muscle. Similar to BAT, inefficient mitochondrial energy conversion could be in play for the skeletal muscle of marsupials. The basal proton leak of isolated skeletal muscle mitochondria in the marsupial *Antechinus flavipes*, however, does not change due to acclimation and therefore is unlikely to contribute to adaptive heat output (Jastroch et al. 2009).

UCP3 is a paralogous protein of UCP1 that is predominantly expressed in skeletal muscle and has been under suspicion to catalyze an inducible proton leak, similar to UCP1. The experimental evidence for such uncoupling activity, however, remains sparse and has not been broadly accepted. Indeed, the function of UCP3 altogether has not been rigorously established. Original claims on UCP3-mediated proton leak in isolated skeletal muscle mitochondria, activated by 4-hydroxynonenal (4-HNE), have been tempered in the meantime (Parker et al. 2008). Also, there is no evidence of physical interaction between the activator 4-HNE and any UCP so far (Jovanovic et al. 2015). In the marsupial *Antechinus flavipes*, 4-HNE induces proton leak of isolated muscle mitochondria selectively in cold-acclimated individuals (Jastroch et al. 2009). 4-HNE activation, however, does not associate with UCP3, given the absence of inhibition of proton leak by guanosine diphosphate, typically used to block UCP activity. Instead, proton leak activation was prevented by carboxy atractylate, a specific inhibitor of the adenine nucleotide translocator (ANT) (Jastroch et al. 2009). In birds, the ANT has been suggested as a candidate mediating muscle NST (Talbot et al. 2004). The protonophoric function of the ANT is mediated by fatty acids (Andreyev et al. 1988, 1989) and in isolated muscle mitochondria of fruit flies, this carrier protein indeed appears to contribute to basal proton leak (Brand et al. 2005). Notably, however, protonophoric function of the ANT has only been shown in isolated mitochondria or with the reconstituted protein in proteoliposomes, but not in intact cells. Thus, it remains unknown whether proton leak through the ANT exists in the living system that depends on a continuous exchange of ADP/ATP, which would compete with proton leak. Another mechanistic proposal for muscle NST is based on inefficient calcium storage in the sarcoplasmic reticulum. Futile cycling of calcium involves the simultaneous activity of the sarcoplasmic/endoplasmic reticulum calcium ATPase (SERCA) to transport calcium into the SR/ER, and the ryanodine receptor to release it into the cytosol, will result in increased ATP hydrolysis and heat production. This mechanism was originally proposed for the retroocular muscle cells of scombroid fishes (Morrissette et al. 2003). In birds, which lack brown fat and UCP1, experimental evidence suggests a similar mechanism based on modified SERCA function, rendering the transporter energy-inefficient by changing the stoichiometry of calcium transport and ATP hydrolysis (Mall et al. 2006). A potential modulator for this is sarcolipin, an associating proteolipid. These potential mechanisms of thermogenesis are critically reviewed by others (Campbell and Dicke 2018). Elegant experimentation would be required to establish these muscle NST mechanisms as significant contributors to systemic thermoregulation in endotherms that lack BAT. In any case, untangling the contribution of muscle shivering vs. non-shivering is challenging, as both processes are located in the same cell and potentially interfere with measurements of one another.

## The unbiased route to NST in marsupials and monotremes

**Fig. 3:**
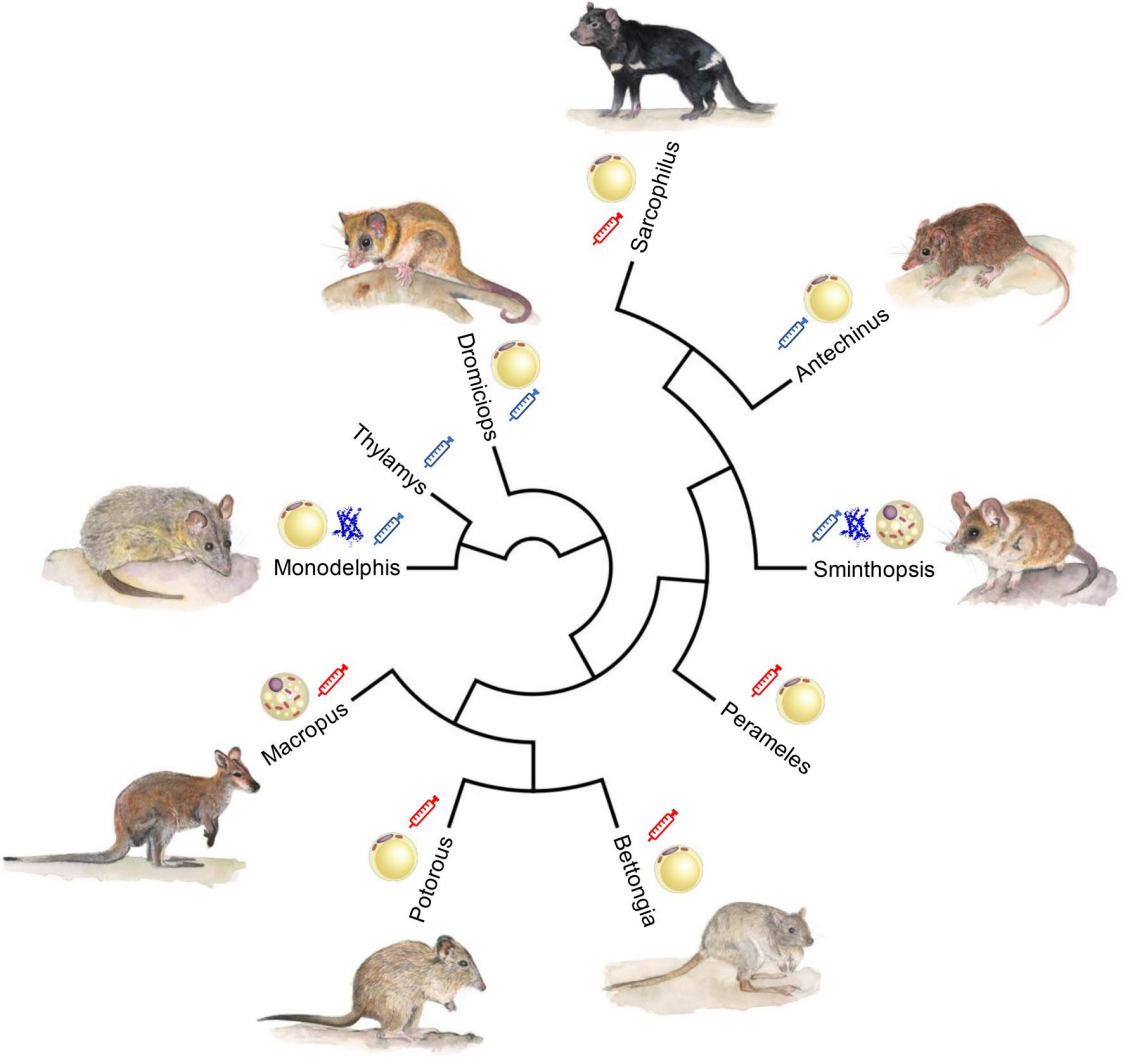
Evidence for adaptive NST, brown fat morphology and UCP1 expression mapped to the marsupial phylogeny. Red syringes denote reports of an increased metabolic rate with NA injection while blue syringes indicate no change in metabolic rate with NA injection compared to saline controls. The ‘brownish’ adipocyte indicates lineages where multilocular adipocytes with high mitochondrial content have been reported while only evidence for unilocular adipocytes was provided for other lineages. The ribbon diagram of UCP1 highlights lineages where UCP1 has been reportedly expressed and localized to adipose tissue. Illustrations of representative species were provided by Chrishanthi Lowe (color figure online)

Considering the lack of a consensus regarding the presence or absence of BAT in marsupials and uncertainties regarding UCP1 expression and function, the primary source of NST in this lineage is still undetermined (Fig. 3), yet it seems that NST could be present given their ability to maintain high body temperatures in the cold. Gene knockout models have been pivotal for the understanding of thermogenic mechanisms among eutherians and should also be applied to the marsupial lineage. The best candidate for such models would likely be *M. domestica* since breeding colonies are relatively common and they have a fairly well-characterized genome. Still, most marsupials are not model laboratory animals and maintenance of them is not trivial. To establish, whether adipocytes at all would be able to generate heat, in vitro experiments with marsupial primary adipocytes could be applied, but are entirely missing in the current literature. Can thermogenesis be recruited in these marsupial adipocytes similar to what is found in murine beige fat and what are the controlling signaling pathways? In this system, the activity of marsupial UCP1 and control of thermogenic energy metabolism could be quantitatively investigated and compared to standard murine adipocytes. In established mammalian cell cultures, the overexpression of marsupial UCP1 opens a window to study protein function and mechanisms, as it has been done previously for mouse UCP1 (Jastroch et al. 2012). Besides thermogenic adipose tissue, the contribution to adaptive NST of other metabolically active organs, such as skeletal muscle, requires better mechanistic experimentation and exploration.

## Concluding remarks

Humans and modern marsupials have been separated by ~ 300 million years of independent evolution. While many consider marsupials to be “primitive” in comparison to eutherians, marsupials are in fact well adapted to cope with environmental challenges of their ecological niches. Indeed, the Virginia opossum (*Didelphis virginianus*) exploits a temperate ecological niche and displays an impressive thermal scope, maintaining a homeothermic milieu (35 °C) at ambient temperatures ranging from 35 °C as low as 3 °C (McManus 1969). Mechanisms of NST have still not been pinpointed in this clade calling for more rigorous experimentation to elucidate how these species may replace shivering thermogenesis with non-shivering means. Whether endothermic mechanisms have evolved independently or are conserved among humans and marsupials, their identification will provide clues of ecophysiological adaptations and perhaps even shed light on our own evolutionary origins and enhance our understanding of how these mechanisms arose and could be therapeutically targeted to treat metabolic diseases such as obesity in the future.
